# Evidence for inbreeding depression in a species with limited opportunity for maternal effects

**DOI:** 10.1002/ece3.1445

**Published:** 2015-03-04

**Authors:** Regina Vega-Trejo, Megan L Head, Michael D Jennions

**Affiliations:** Division of Evolution, Ecology and Genetics, Research School of Biology, The Australian National UniversityActon, ACT, 2601, Australia

**Keywords:** Lecithotrophic, maternal investment, offspring fitness, relatives

## Abstract

It is often assumed that mating with close relatives reduces offspring fitness. In such cases, reduced offspring fitness may arise from inbreeding depression (i.e., genetic effects of elevated homozygosity) or from post-mating maternal investment. This can be due to a reduction in female investment after mating with genetically incompatible males (“differential allocation”) or compensation for incompatibility (“reproductive compensation”). Here, we looked at the effects of mating with relatives on offspring fitness in mosquitofish, *Gambusia holbrooki*. In this species, females are assumed to be nonplacental and to allocate resources to eggs before fertilization, limiting differential allocation. We looked at the effects of mating with a brother or with an unrelated male on brood size, offspring size, gestation period, and early offspring growth. Mating with a relative reduced the number of offspring at birth, but there was no difference in the likelihood of breeding, gestation time, nor in the size or growth of these offspring. We suggest that due to limited potential for maternal effects to influence these traits that any reduction in offspring fitness, or lack thereof, can be explained by inbreeding depression rather than by maternal effects. We highlight the importance of considering the potential role of maternal effects when studying inbreeding depression and encourage further studies in other Poeciliid species with different degrees of placentation to test whether maternal effects mask or amplify any genetic effects of mating with relatives.

## Introduction

Mating with close relatives often reduces offspring fitness (Keller and Waller 2002). This can take the form of a reduction in offspring birth weight, survival, or reproductive success, as well as resistance to disease, predation, and environmental stress (Keller and Waller 2002; Frommen et al. 2008). The decrease in offspring fitness resulting from mating with close relatives is often attributed to inbreeding depression (Charlesworth and Charlesworth 1987; Falconer and Mackay 1996). Inbreeding depression results from an increase in the levels of homozygosis (Keller and Waller 2002; Frommen et al. 2008) and has been explained by two main hypotheses. The overdominance hypothesis, where heterozygotes, which are assumed to be superior to homozygotes, decrease in frequency, and the partial dominance hypothesis where the unmasking of deleterious recessive alleles due to greater homozygosity reduces fitness (Charlesworth and Charlesworth 1987). However, inbreeding depression is not the only explanation for differences in offspring fitness when mating with close relatives rather than unrelated individuals.

Maternal investment in offspring in response to male traits is known to have important effects on offspring phenotypes (Kindsvater and Alonzo 2014). This means that variation in offspring traits, particularly those expressed early in life, may result from variation in maternal investment (i.e., maternal effects) rather than being solely attributable to offspring genotype. Mothers can differentially allocate resources into offspring to maximize their fitness (Sheldon 2000). This is widely associated with greater maternal investment into offspring sired by more attractive males, who possess generally preferred traits (e.g., large ornaments; Arct et al. 2010; Horvathova et al. 2012). It follows that differential allocation by females may also be influenced by the relatedness of their mating partner (Lihoreau et al. 2008) as genetically similar males are generally considered to be less attractive mates because of the potential costs of inbreeding (Tregenza and Wedell 2000). Females may therefore be expected to reduce investment in offspring that are sired by closely related males (e.g., Sardell and DuVal 2014). Alternatively, females could partially compensate for the lower quality of their offspring by providing more resources when mating to nonpreferred or genetically incompatible mates (Ratikainen and Kokko 2010). If present, maternal effects may enhance (for differential allocation) or mask (for reproductive compensation) the potentially negative genetic effects of mating with a relative.

Early life-history traits such as embryo survival, number, quality, and the viability of offspring (Bernasconi et al. 2004; Frommen et al. 2008) are closely related to fitness (DeRose and Roff 1999; Janicke et al. 2014) and, as such, often suffer from inbreeding depression (Roff 1998; DeRose and Roff 1999). However, these are the same traits that are most likely to be influenced by maternal effects (Wolf and Wade 2009; Kindsvater and Alonzo 2014). Consequently, it is important for studies investigating how mating with relatives influences offspring performance to consider, and ideally control for, maternal effects to avoid potentially inaccurate measures of inbreeding depression.

Here, we examine the effects of mating with relatives on offspring fitness in mosquitofish (*Gambusia holbrooki*), a species with limited opportunity for post-mating maternal effects. They are small fish that live in streams and ponds (Pyke 2005) with seasonally fluctuating water levels, so they are often exposed to stochastic reductions in population size, especially during dry seasons (Scribner et al. 1992; Griffiths and Magurran 1997). This makes them vulnerable to the risk of inbreeding. Furthermore, mosquitofish are lecithotrophic (i.e., allocate resources for embryo development to eggs before fertilization), which limits the opportunity for females to differentially allocate resources toward offspring after mating (i.e., matrotrophy; Ojanguren et al. 2005; Pollux et al. 2014). There is limited evidence of transfer of nutrients such as amino acids and metals in other species of mosquitofish (Marsh-Matthews et al. 2005, 2010; Cazan and Klerks 2014) that suggests post-fertilization transfer from mother to embryos. Although this means that there is the potential for maternal effects to confound those directly due to inbreeding depression, the lack of evidence for an increase in offspring mass between the egg and birth stage strongly suggests that transfer of nutrients does not generally occur in *Gambusia holbrooki* (Pollux et al. 2014).

We looked at the effects of mating with a sibling on several reproductive and early life-history traits. We examined offspring number, offspring size, gestation period, and early offspring growth. If we assume, based on the lack of evidence for matrotrophy, that eggs are fully provisioned prior to mating, we predicted that genetic effects of mating with relatives would most likely influence the number of offspring (via effects during fertilization or embryo development), as well as their size at birth and their growth after birth. On the other hand, we predicted that maternal effects are likely to influence the proportion of females breeding and gestation time (i.e., females can determine if and when to fertilize eggs).

## Materials and Methods

### Origin and maintenance of fish

Our laboratory stock of mosquitofish originated from 151 wild-caught females collected in Canberra, Australia in February and March 2013. F_1_ generation offspring were kept in single sex tanks under a 14:10 h photoperiod at 28°C and fed ad libitum with *Artemia nauplii* and commercial flakes.

### Experimental design

**Figure 1:**
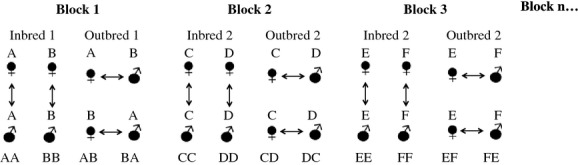
Block design for mating F_2_ families to create inbred vs outbred fish. Each block involved between one and four full sisters and one male per cross-type from two families (A and B in block 1, C and D in block 2, E and F in block 3, and so on). Arrows indicate matings.

To create our parental generation, we set up 150 unique male–female pairs that were randomly created from the F_1_ laboratory stock (described above). From these, we obtained 58 outbred F_2_ full-sib families that were used to examine the effects of mating with relatives on female reproductive effort and early life offspring performance. We used a fully balanced block design that involved mating individuals from two families (e.g., A and B). Brothers and sisters from full-sibling families were paired to create inbred offspring (AA and BB) and outbred offspring by the reciprocal crossings of males and females from each family (AB and BA; Fig.1). We set up multiple females (one to four full sisters) per cross-type (AA, AB, BA, BB). Within each block, the same potential number of females contributed to each cross-type. Only one male contributed to each cross-type so that within each block the offspring of each cross-type were either full siblings or paternal half siblings. Males and females were placed together for 1 week to allow mating. Females were then placed in individual 1-L tanks and allowed 6 weeks to give birth. They were checked for offspring twice daily. We set up 29 blocks yielding a maximum total of 58 inbred families and 58 outbred families. We recorded the age and size (standard length, SL in mm) of each female on the day she gave birth, the gestation time, the number of offspring, the size of offspring at birth, and their size 1 week later. To measure female size, females were anaesthetized by submersion in ice-cold water for a few seconds to reduce movement and then photographed alongside a microscopic ruler (0.1 mm gradation). To measure offspring size, fry were placed in a plastic dish (27 × 27 mm) with 2 mm depth of water to restrict movement and a scale at the bottom. All offspring were photographed within 18 h of birth.

### Statistical analysis

We tested for a difference in reproductive success between females mating with a related or an unrelated male by comparing the proportion that gave birth within 6 weeks of the mating period using a chi-squared test. When testing for an effect of mating with relatives on gestation time and the number of offspring produced, we only included first broods by females that gave birth during the first 6 weeks. This avoids any confounding effect of a change in brood size with brood order (Larsen et al. 2011). These analyses were based on a single value per brood. To test for an inbreeding effect on size at birth and growth rates (size at 1 week of age – size at birth), we included the data from each individual offspring that the female gave birth to. Cross-types AA and BB were classified as inbreeding, while AB and BA were classified as outbreeding.

### Female reproductive effort

We used generalized linear mixed-effect models (GLMM) with Gaussian error to test for fixed effects of treatment (related or unrelated male), female age, and female size on gestation time, number and size of offspring, and the growth rate of offspring with the *lmer* function using the *lme4* package in R 3.0.2 software (R Development Core Team 2012). We included the female's family identity as a random effect when testing for effects on gestation time and number of offspring. We included the female's individual identity as a random factor when testing offspring size and growth (as we measured multiple fry per female). We treated maternal age and size as independent predictors because they were uncorrelated (*r = *−0.027, *P *=* *0.716, *N* = 179; age range: 82–141 days, size: 22.76–31.25 mm).

### Inbreeding coefficient

We calculated the standardized coefficient of inbreeding δ (Lande and Schemske 1985) as the percentage change with inbreeding: (outbred trait value – inbred trait value) / outbred trait value. A negative value indicates that inbred individuals had a larger value for the trait, interpretation of which depends on the direction of selection on the trait.

## Results

There was no difference in the proportion of females producing broods when mated with either a related or unrelated male. From 162 females that mated with their brother, 79.6% gave birth, while from 147 females mated with an unrelated male, 77.5% gave birth (χ^2^* = *0.198, df* = *1, *P *=* *0.656). From 309 females that could have produced broods, 199 were used for analyses of first broods produced within 6 weeks of mating (112 mated with a brother; 87 with an unrelated male).

### Female reproductive effort

**Figure 2:**
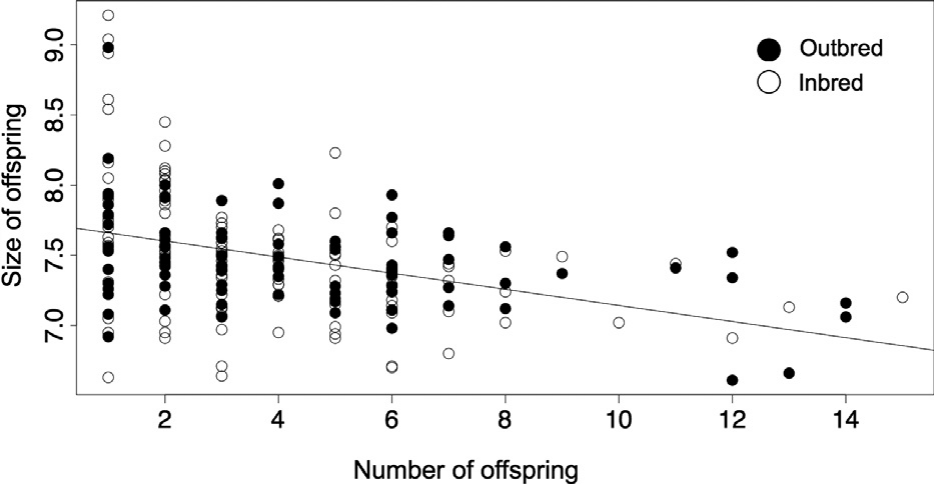
The association between number and size of outbred and inbred offspring.

The number of offspring a female gave birth to (range: 1- 15) was affected by whether or not she mated with a related male (Fig.2). Females mated to their brother gave birth to significantly fewer offspring than those mated to an unrelated male (an inbreeding coefficient of δ* = *14.5%; Table1). The number of offspring in the brood was significantly negatively related to the female's age, but significantly positively related to her size (Table1).

**Table 1:** Results of GLMs (Gaussian error) for the response variables: gestation time, number of offspring, size of offspring, and growth of offspring of females mated to related and unrelated males. Inbreeding coefficient (% change with inbreeding). Bold values represent significant values

Response	Predictor	*β*	SE	df	*t*	*P*	Mean ± SE (*N*)		δ
							Inbred	Outbred	
Gestation time (days)	Intercept	28.756	17.224	101.150	1.669	0.098	33.67 ± 0.794 (112)	32.33 ± 0.883 (87)	−4.145
	Treatment	1.228	1.204	159.180	1.020	0.309			
	Female size	0.230	0.532	132.770	0.432	0.667			
	Female age	−0.028	0.083	70.880	−0.343	0.733			
Number of offspring	Intercept	−4.941	6.125	112.740	−0.807	0.422	3.83 ± 0.256 (112)	4.48 ± 0.344 (87)	14.509
	Treatment	−1.003	0.404	154.160	−2.481	**0.014**			
	Female size	0.671	0.186	143.070	3.599	**<0.001**			
	Female age	−0.065	0.030	77.000	−2.180	**0.032**			
Size of offspring (mm)	Intercept	7.030	0.783	101.800	8.975	<0.001	7.352 ± 0.029 (212)	7.368 ± 0.016 (590)	0.217
	Treatment	0.021	0.069	135.200	0.310	0.757			
	Female size	0.003	0.024	121.700	0.138	0.890			
	Female age	0.003	0.004	85.940	0.763	0.448			
Growth of offspring (mm in first week)	Intercept	3.104	1.164	99.150	2.666	0.009	3.633 ± 0.045 (172)	3.560 ± 0.028 (501)	−2.050
	Treatment	0.106	0.094	140.540	1.128	0.261			
	Female size	0.024	0.036	114.280	0.680	0.498			
	Female age	−0.003	0.005	83.500	−0.589	0.557			

In contrast, we found no evidence that mating with related males affected the gestation time of females, the size of offspring at birth (range: 6.61–9.21 mm), or early offspring growth. Nor did we find any effect of female size or age on any of these traits. Further, we found no repeatable difference in gestation time among families (Table1).

## Discussion

Variation in traits expressed in offspring can be attributable to both parental effects and offspring genotype. For example, life-history traits related to female reproductive effort are a maternal character but they can also affect offspring fitness (Bernardo 1996; Fischer et al. 2006). When assessing whether mating with relatives causes inbreeding depression, maternal effects from differential allocation or reproductive compensation could exacerbate or mask potential genetic effects. In the present study, we found that mating with a relative (full sibling) in *Gambusia holbrooki* significantly reduced the number of offspring at birth (δ* = *14.5%). There was, however, no significant decrease in the likelihood of breeding, no increase in gestation time (δ* = *4.2%), and no reduction in the size (δ* = *0.2%) or growth (δ* = *2.1%) of the resultant offspring. Given the reproductive physiology of *G. holbrooki* (fully yolked eggs are produced prior to mating), there is no obvious mechanism for post-mating maternal effects on offspring size or growth, and maternal effects on offspring number and gestation time seem unlikely. It has, however, been suggested that mosquitofish are incipient matrotrophic rather than lecithotrophic organisms based on transfer of metals from mothers to offspring (Cazan and Klerks 2014), so we cannot definitively exclude the possibility that there are subtle maternal effects. Nonetheless, the decline in offspring dry weight from the egg to birth stage in *G. holbrooki* suggests that there is no transfer of nutrients to offspring (Pollux et al. 2014).

The smaller brood size of females mated to a related rather than an unrelated male has several potential explanations. First, sperm allocation toward related and unrelated females might differ (Firman and Simmons 2008; Lewis and Wedell 2009). However, it is unlikely that this explains our findings because males did not choose between females, and previous studies on mosquitofish (Head et al.*, in press*) and more generally (Barry and Kokko 2010) show that males are rarely choosy when encountering females sequentially. Further, even very low sperm transfer is still likely to provide sufficient sperm to fertilize a full clutch (Bisazza and Marin 1991; Johnson et al. 2010). Second, females might decide not to fertilize all their eggs when mating with males of low compatibility (e.g., Olsson et al. 1996; Birkhead 1998). This is unlikely for several reasons: (1) Our experimental design reduced the potential for choice – females were virgins and previous work on Poeciliids has shown that virgins are not choosy with respect to mate quality (Pitcher et al. 2003), (2) There is little evidence of mate choice for unrelated males in Poeciliids (e.g., Pitcher et al. 2008; Ala-Honkola et al. 2010), but see (Kelley et al. 1999; Zajitschek and Brooks 2008) for studies showing male mate preferences based on familiarity and (Hain and Neff 2007) showing kin recognition in Poeciliids), and (3) If females differentially used sperm, this should increase their gestation time, and/or affect the proportion of females breeding. This did not occur. Females cannot provision eggs after fertilization, and lack superfetation (Ojanguren et al. 2005; Pollux et al. 2014), so there is no immediate benefit of discriminating against a related male's sperm (e.g., Pitcher et al. 2008). In short, there is no obvious adaptive explanation why females would partially fertilize a clutch.

Third, the most plausible explanation for females having fewer offspring when mated with related males is reduced fertilization success (i.e., low sperm survival due to sperm–female tract or egg interactions) and/or inbreeding depression lowering embryo survival (Pitcher et al. 2008; Johnson et al. 2010). In general, the evidence for a negative effect of mating with a related male-on-female reproductive effort is inconclusive: some studies report fewer offspring or eggs (e.g., Pitcher et al. 2008; Johnson et al. 2010), but others do not (e.g., Simmons et al. 2006; Ala-Honkola et al. 2009). However, based on studies of other Poeciliids, inbreeding depression for embryo viability is most likely to explain why *G. holbrooki* had fewer offspring after a full-sib mating (Pitcher et al. 2008; Johnson et al. 2010).

Offspring size at birth is under directional selection as larger offspring tend to be more competitive and survive better in stressful environments (Smith and Fretwell 1974; Simmons and Garcia-Gonzalez 2007). Larger offspring also tend to become adults with above average reproductive success (e.g., Czesak and Fox 2003). We did not, however, find any evidence of inbreeding reducing offspring size at birth or post birth growth, even though this should occur if higher homozygosity reduces the physiological efficiency with which offspring convert resources (i.e., egg yolk then *Artemia*) into body mass. One explanation for a lack of inbreeding depression is that offspring with bad genetic combinations died before birth. This explanation is also consistent with fewer offspring being born to females who mated with a brother.

In our experiment, males and females were allowed 1 week to interact and mate. We predicted that if females avoid mating with related males that those paired with their brother would take longer to mate and/or refrain from fertilizing their eggs and therefore would take longer to give birth. This did not occur. There is conflicting evidence for effects of mating with relatives on gestation time in Poeciliids: Some studies show that it increases (e.g., Pitcher et al. 2008), while others show no difference in gestation time (e.g., Ala-Honkola et al. 2009). Further experiments measuring egg fertilization following artificial insemination might yield more information about the mechanism, if any, by which females reduce the likelihood of inbreeding.

## Conclusions

Studies often report reduced reproductive performance of females mating with related males and attribute this to inbreeding depression (i.e., genetic effects). These studies, however, almost always ignore the potential role of postmating maternal effects in response to the identity of their mating partner. Here, we show a reduction in the number of offspring produced when females mated with a full sibling in the mosquitofish, a species that has limited opportunity to influence this trait via maternal effects. Furthermore, there was no difference between females mated to related or unrelated males in traits that we expected to be influenced by maternal effects (gestation time and whether they breed) or in traits that are unlikely to be affected by maternal affects (offspring birth size and growth). A comparative study measuring inbreeding effects in species that vary in their ability to alter offspring traits via post-mating maternal effects is needed. We suggest that Poeciliids are an ideal group in which to conduct the requisite empirical studies because: (1) closely related species vary substantially in their level of placentation (Pollux et al. 2014), hence ability to adjust provisioning of nutrients to offspring, depending on the relatedness of their mate; (2) the risk of inbreeding seems to have played a role in mate choice in some Poeciliids (e.g., Zajitschek and Brooks 2008) so an adaptive phenotypically plastic maternal response based on relatedness to males with whom they mate is plausible.

## Data Accessibility

Data will be deposited in Dryad upon acceptance.
